# Sensitivity and specificity of International Classification of Diseases algorithms (ICD-9 and ICD-10) used to identify opioid-related overdose cases: A systematic review and an example of estimation using Bayesian latent class models in the absence of gold standards

**DOI:** 10.17269/s41997-024-00915-4

**Published:** 2024-07-31

**Authors:** Fiston Ikwa Ndol Mbutiwi, Ayekoe Patrick Junior Yapo, Serge Esako Toirambe, Erin Rees, Rebecca Plouffe, Hélène Carabin

**Affiliations:** 1 Faculté de médecine vétérinaire, Université de Montréal, Saint-Hyacinthe, Québec Canada; 2 Département de médecine sociale et préventive, École de santé publique, Université de Montréal, Montréal, Québec Canada; 3 Faculty of Medicine, University of Kikwit, Kikwit, Kwilu Democratic Republic of the Congo; 4 National Microbiology Laboratory, Public Health Agency of Canada, Saint-Hyacinthe, Québec Canada; 5 Centre for Surveillance and Applied Research, Health Promotion and Chronic Disease Prevention Branch, Public Health Agency of Canada, Ottawa, Ontario Canada; 6 Groupe de recherche en épidémiologie des zoonoses et santé publique (GREZOSP), Saint-Hyacinthe, Québec Canada; 7 Centre de recherche en santé publique de l’Université de Montréal et du CIUSSS du Centre-sud-de-l’île-de-Montréal (CReSP), Montréal, Québec Canada

**Keywords:** Opioid overdose, Sensitivity, Specificity, International Classification of Diseases, Bayesian analysis, Systematic review, Surdose aux opioïdes, sensibilité, spécificité, Classification Internationale des Maladies, analyse Bayésienne, revue systématique

## Abstract

**Objectives:**

This study aimed to summarize validity estimates of International Classification of Diseases (ICD) codes in identifying opioid overdose (OOD) among patient data from emergency rooms, emergency medical services, inpatient, outpatient, administrative, medical claims, and mortality, and estimate the sensitivity and specificity of the algorithms in the absence of a perfect reference standard.

**Methods:**

We systematically reviewed studies published before December 8, 2023, and identified with Medline and Embase. Studies reporting sufficient details to recreate a 2 × 2 table comparing the ICD algorithms to a reference standard in diagnosing OOD-related events were included. We used Bayesian latent class models (BLCM) to estimate the posterior sensitivity and specificity distributions of five ICD-10 algorithms and of the imperfect coroner’s report review (CRR) in detecting prescription opioid–related deaths (POD) using one included study.

**Results:**

Of a total of 1990 studies reviewed, three were included. The reported sensitivity estimates of ICD algorithms for OOD were low (range from 25.0% to 56.8%) for ICD-9 in diagnosing non-fatal OOD-related events and moderate (72% to 89%) for ICD-10 in diagnosing POD. The last included study used ICD-9 for non-fatal and fatal and ICD-10 for fatal OOD-related events and showed high sensitivity (i.e. above 97%). The specificity estimates of ICD algorithms were good to excellent in the three included studies. The misclassification-adjusted ICD-10 algorithm sensitivity estimates for POD from BLCM were consistently higher than reported sensitivity estimates that assumed CRR was perfect.

**Conclusion:**

Evidence on the performance of ICD algorithms in detecting OOD events is scarce, and the absence of bias correction for imperfect tests leads to an underestimation of the sensitivity of ICD code estimates.

**Supplementary Information:**

The online version contains supplementary material available at 10.17269/s41997-024-00915-4.

## Résumé

**Objectifs:**

Cette étude avait pour objectifs de recenser les estimations de la validité des codes de Classification Internationale des Maladies (CIM) à diagnostiquer les cas de surdose aux opioïdes (SDO) chez des patients en utilisant les données de salles d’urgence, services médicaux d’urgence, hospitalisations, soins ambulatoires, services administratifs, demandes de remboursement de frais médicaux, ainsi que de mortalité, et d’estimer la sensibilité et la spécificité d’algorithmes utilisant la CIM en l’absence d’un test de référence parfait.

**Méthodes:**

Nous avons examiné systématiquement les études publiées avant le 8 décembre 2023, et identifiées dans Medline et Embase. Les études rapportant suffisamment de détails permettant de recréer un tableau 2 × 2 comparant les algorithmes de la CIM à un test de référence pour le diagnostic d’événements liés aux SDO ont été incluses. Les données d’une étude éligible ont été utilisées pour estimer, avec des modèles Bayésiens de classes latentes (MBCL), les distributions a posteriori de la sensibilité et de la spécificité de cinq algorithmes de la CIM-10 et du test imparfait de révision du rapport du coroner (RRC) dans la détection des décès liés aux opioïdes de prescription (DOP).

**Résultats:**

Trois parmi les 1 990 études examinées ont été retenues. Les estimations rapportées de la sensibilité des codes CIM étaient faibles (variant de 25,0 % à 56,8 %) pour CIM-9 dans le diagnostic des événements liés aux SDO non-fatales dans une étude, et modérées (72 % à 89 %) pour CIM-10 dans le diagnostic des DOP dans une autre étude. La dernière étude incluse combinait des codes CIM-9 pour les cas non-fatals et fatals et CIM-10 pour les cas fatals et démontrait des estimations de sensibilité élevées (c.à.d. supérieures à 97 %). Les estimations de la spécificité étaient bonnes à excellentes dans les trois études. Les estimations de la sensibilité des algorithmes de la CIM-10 corrigées pour les erreurs de classification pour les décès liés aux opioïdes, obtenues à partir de nos MBCL, étaient systématiquement plus élevées que celles rapportées et qui supposaient que RRC était un test parfait.

**Conclusion:**

Les évidences sur la performance des algorithmes de la CIM dans la détection des cas de SDO sont rares, et l’absence de correction de biais pour des tests diagnostiques imparfaits conduit à une sous-estimation de la sensibilité des codes de la CIM.

## Introduction

Opioids are highly effective to relieve pain, making them one of the most widely prescribed, yet deadly, drugs used globally. Opioids are used to reduce pain in a variety of conditions, including cancer, rheumatic diseases, and surgery, and were estimated to be used by 60 million people, representing 1.2% of the global population, in 2020 (Anastasiou & Yazdany, 2022; Dalal & Bruera, 2019; Fiore et al., 2022). However, opioids are responsible for more than two thirds (69%) of drug overdose deaths worldwide, making them the most lethal drugs (Penington Institute, 2022; UNODC, 2022). Opioid overdose (OOD) is an acute clinical condition characterized by a symptomatic triad of unconsciousness, myotic pupils, and respiratory depression, which accounts for a large proportion of opioid-related deaths (Parthvi et al., 2019). OOD can occur in various settings including misuse, chronic pain management, and accidental exposure as well as for inpatient use (Algera et al., 2021; Bateman et al., 2023; Khanna et al., 2020; Madadi et al., 2013), and may involve pharmaceutical or non-pharmaceutical opioids (Ogeil et al., 2018; Velagapudi & Sethi, 2023).

The number of deaths due to OOD is increasing in Canada. It is estimated that from the beginning of January 2016 to June 2023, 40,642 deaths associated with the opioid crisis were reported (PHAC, 2023). From January to June 2023, 3970 deaths apparently related to opioid intoxication occurred. This corresponds to 22 deaths per day, much higher than the 8 and 12 deaths reported in 2016 and 2018, respectively. The provinces of British Columbia, Alberta, and Ontario reported 89% of these deaths during the first 6 months of 2023. This public health crisis is of greatest concern in young to middle-aged (29–59 years old) men who represented 72% of all deaths during the same period, with fentanyl being the leading opioid molecule accounting for 84% of all OOD death cases across Canada (PHAC, 2023).

International Classification of Diseases (ICD) is the most widely used system for recording morbidity and mortality data in electronic medical records (Lutomski et al., 2012; Walker et al., 2012; Zhu et al., 2022). However, it has been reported that the assignment of ICD codes by medical coders does not always accurately reflect the diagnoses made by the clinicians or the procedures applied by medical staff (Liang et al., 2011; McGrew et al., 2020; Sarrazin & Rosenthal, 2012). Such misclassification may lead to biased estimates of health condition prevalence based on administrative data (McGrew et al., 2020). This has been highlighted for opioid use disorder in particular, a chronic relapsing disorder involving the use of opiates, for which limitations of ICD-9/10 codes including misclassification, low sensitivity, and underestimation of prevalence have been reported (Hallgren et al., 2020; Ranapurwala et al., 2023; Roland et al., 2016; Zhu et al., 2022). In the case of OOD, misclassification of ICD codes may be introduced from incorrect or incomplete documentation by treating clinicians, or alternatively, when other drugs are incriminated as potential causes of the patient’s medical visit or death. ICD codes for fatal or non-fatal OOD cases/poisoning are numerous, and their assignment or processing, especially when multiple substances are involved, can be challenging. In addition, assessment of the validity of ICD codes to correctly classify many conditions, including OOD, is often determined by comparing them to manual review of medical records which is considered a reference standard (Chartash et al., 2019; Green et al., 2017, 2019; Ranapurwala et al., 2023; Rowe et al., 2017; Ward et al., 2023). However, manual chart review is also likely imperfect since reviewers’ expertise and training as well as interpretations of what is written on the medical chart may differ (Gladstone et al., 2016; Green et al., 2017, 2019). Furthermore, the presence of the drug could have been tested too late and therefore not reported even though it was the cause of the medical visit, leading to missing cases through medical record review (and ICD), adding uncertainty to the reported validity of the ICD codes.

Statistical methods, such as Bayesian latent class models (BLCM), model test sensitivity and specificity, as well as the true disease prevalence, as unobserved latent parameters while incorporating prior information on their performance when available (Angelidou et al., 2014; Arango-Sabogal et al., 2018; Berman et al., 2019; Branscum et al., 2005). To date, no study has focused on estimating the performance of ICD codes to classify OOD cases assuming no gold standard exists. Adequate planning of the response to the ever-growing opioid crisis requires accurate knowledge of the extent of OOD across the country and through time, which implies that available medical administrative data provide valid information on the frequency of OOD (Walker et al., 2012). With the increasing use of ICD codes in the monitoring of drug overdose events (Coben et al., 2010; Rowe et al., 2017; Slavova et al., 2014; Xiang et al., 2012), it is imperative that a comprehensive examination of the available evidence be done to determine whether ICD codes are a good tool for estimating and comparing OOD frequency estimates.

The goal of this study was to systematically review published studies evaluating the validity of ICD algorithms compared to any another source of information in diagnosing of OOD events among data obtained from emergency departments, emergency medical services, inpatient, outpatient, administrative, medical claims, and death reports and estimate the misclassification-adjusted sensitivity and specificity of ICD algorithms in identifying OOD-related events.

## Methods

This systematic review was conducted according to the Preferred Reporting Items for Systematic Reviews and Meta-Analyses (PRISMA) checklist (Moher et al., 2009) (Online Resource 1). The study protocol was registered with PROSPERO (registration number CRD42023408943), and is available from https://www.crd.york.ac.uk/prospero/display_record.php?RecordID=408943.

### Study search

The study search strategy was developed by a trained health sciences librarian (SF) at Université de Montréal in collaboration with the researchers, and was implemented in MEDLINE and EMBASE databases. Studies published in English, French, Italian, or Spanish were eligible. An initial search conducted by SET on May 6, 2023, resulted in a limited number of relevant articles and only one deemed eligible for inclusion. A new search was run on December 13, 2023, by FINM adding more general keywords to the previous search strategy. Both full search strategies are provided in Online Resource 2. We also performed snowball manual searches for each study eligible for inclusion in our review as well as from review article in an attempt to identify any additional relevant studies that our search strategy may have missed.

### Study selection

All identified studies were uploaded into Covidence web-based collaboration software platform (Veritas Health Innovation Ltd, Melbourne, Australia) for the screening phase of the review.

First, titles and abstracts of the identified papers were screened using the following inclusion criteria: (1) the study populations were individuals who had sought emergency medical services (ambulances) and emergency room visits, and were hospitalized or seen as outpatients, for whom medical claims were created or who died; (2) the outcome of interest was either fatal or non-fatal OOD-related events; and (3) participants were evaluated with at least two diagnostic tests to identify OOD-related events. We excluded editorials, comments, literature reviews, and letters to the editor of journals. The titles and abstracts were screened by two independent reviewers (SET and FINM for initial search, and FINM and APJY for the second search).

Second, the full texts of articles deemed eligible after screening were independently revised by the same two reviewers, using the following inclusion criteria: (1) ICD-9 or ICD-10 algorithms were used as one of the diagnostic tests for OOD-related events; and (2) the data presented in the article allowed the building of a two-by-two table (presence/absence of OOD-related events) of agreement of ICD algorithms compared to a reference standard or another test. We excluded studies using a mixture of both ICD and other classification system codes as an index test which did not allow to isolate classification by ICD code only. Any disagreement between the reviewers was resolved by discussion until consensus was reached.

### Data extraction

Two authors (FINM and APJY) independently extracted relevant data from included studies using a standardized form for study evidence synthesis. Discrepancies and unclear issues were addressed in discussion with a third reviewer (HC). In the event of missing data, we contacted the authors of the study to obtain additional information. Extracted data included study setting, study population and its demographic characteristics, data sources used, study methodology, outcomes and measurement times, definition of the outcome according to the ICD codes and the reference standard used, agreement between the two tests with the contents of the 2 × 2 table, and measures of validity of ICD codes versus the reference standard.

### Study quality assessment

The two primary reviewers (FINM and APJY) independently assessed the risk of bias in the selected studies using the QUADAS-2 (Quality Assessment of Diagnostic Accuracy Studies) tool (Whiting et al., 2011) focusing on its four key domains related to the selection of study, the conduct or interpretation of the index criterion (i.e. ICD codes) as well as of the reference standard or other test used as a comparison, and finally, the way in which missing data were handled, and their potential impact on the study results. The risk of bias was considered to be “low”, “high”, or “unclear” based on the answers provided to the signaling questions in each of the four QUADAS-2 domains. If the answers to all signaling questions for a domain were “yes”, then risk of bias was judged low. If any signaling question was answered “no”, then the risk of bias was judged high. If any signaling question was answered “unclear”, then the risk of bias was judged “unclear”. The second signaling question of domain 3, relating to the index test threshold, was not considered since the outcome of interest of the review was binary (presence/absence of OOD event). Both reviewers also assessed the applicability of each included study to our review, and concerns regarding applicability for the first three domains of QUADAS-2 tool were judged “low”, “high”, or “unclear” (Whiting et al., 2011). Areas of disagreement among the two reviewers about the potential for unbiasedness of studies or applicability concerns were resolved through discussion with a third reviewer (HC). The instructions used to answer the signaling questions of QUADAS-2 tool were adapted from the work by McGrew et al. (2020) (Online Resource 3).

### Data synthesis and analysis

Characteristics of selected studies were described and presented in a table. No meta-analysis was performed due to small number (*n* = 3) and heterogeneity of studies included.

We used BLCM to estimate misclassification-adjusted sensitivity and specificity of ICD-10 algorithms and coroner’s report review (CRR) for the one study (Gladstone et al., 2016) with low risk of bias in all four QUADAS-2 domains, as recommended (Whiting et al., 2011).

Traditionally, the sensitivity and specificity of a diagnostic test are assessed against a reference test assumed perfect to classify disease status (i.e. without error) (Cheung et al., 2021; Collins & Huynh, 2014). In cases where the reference test is imperfect, estimates of sensitivity and specificity can be biased (Collins & Huynh, 2014), resulting in an inaccurate estimate of disease prevalence. BLCM can be used to adjust for such misclassification error by considering each subject’s disease status as latent (existing but unobserved), and by estimating the probability that each subject has the disease conditional on an observed diagnostic test result, and prior information on test accuracy and disease prevalence (Berman et al., 2019; Cheung et al., 2021). One key feature of BLCM is the availability of reliable prior values, and the justification for their distribution, since prior information can influence the results (Kostoulas et al., 2017).

In the study by Gladstone et al. (2016), five ICD-10 algorithms were each used as an independent index test against CRR as reference standard, to identify prescription opioid–related deaths (POD) among all drug- or alcohol-related deaths. CRR was considered an imperfect test given the lack of standards for coroners’ forensic investigation or classification of deaths, and the absence of a nationally recognized training program or credentialing system for coroners in Canada (Kelsall & Bowes, 2016). Thus, the certification of some deaths by different coroners may not be uniform and may be subject to misclassification (McLean, 2017; Parai et al., 2006).

We were unable to find prior information on the performance of either CRR or ICD-10 codes in identifying POD. Therefore, we created 12 scenarios combining uniform prior distributions for the sensitivity and specificity of the CRR and ICD-10 algorithms. We present two extreme scenarios in terms of prior values. Scenario 1 used more informative priors with uniform distributions of (0.75–1.00) for the sensitivity and of (0.90–1.00) for the specificity of the CRR and all ICD-10 algorithms. Scenario 2 used vague uniform distributions of (0.00–1.00) for the sensitivity and the specificity of the CRR and all ICD-10 algorithms. The other ten sets of priors used gave similar results and are not presented. In all scenarios, the prior distribution of the true prevalence was loosely based on a study conducted in Finland between 2000 and 2004 among medico-legally autopsied deaths where a blood analysis showed evidence of drug use (Lahti et al., 2009). The study found that 10.2% of these deaths had evidence of opiates in their blood in 2004. The study population of Gladstone et al.’s study (2016) was less at risk of opioid-related deaths because it included all deaths linked to drugs and alcohol and the OOD were limited to prescription opiates only. Therefore, we assumed that the prior for the true prevalence follows a uniform distribution between 0% and 10.2%.

We assumed conditional independence between the two tests as information on prescription opioid–related mortality diagnoses by ICD-10 codes and by CRR were extracted from two separate databases and used different criteria to come up with this diagnosis. The BLCM were run for 600,000 iterations with 3000 burn-in. We report here the median and 95% Bayesian credible intervals (95%BCI) of the estimated posterior distributions for the sensitivity and specificity of the two tests. We assessed convergence for Markov chain Monte Carlo sampling by visual inspection of the trace plots and using the Gelman-Rubin diagnostic criterion (Gelman et al., 1996). Analyses were performed in R using JAGS (Just Another Gibbs Sampler) software (codes presented in Online Resource 4).

## Results

### Study selection

**Fig. 1:**
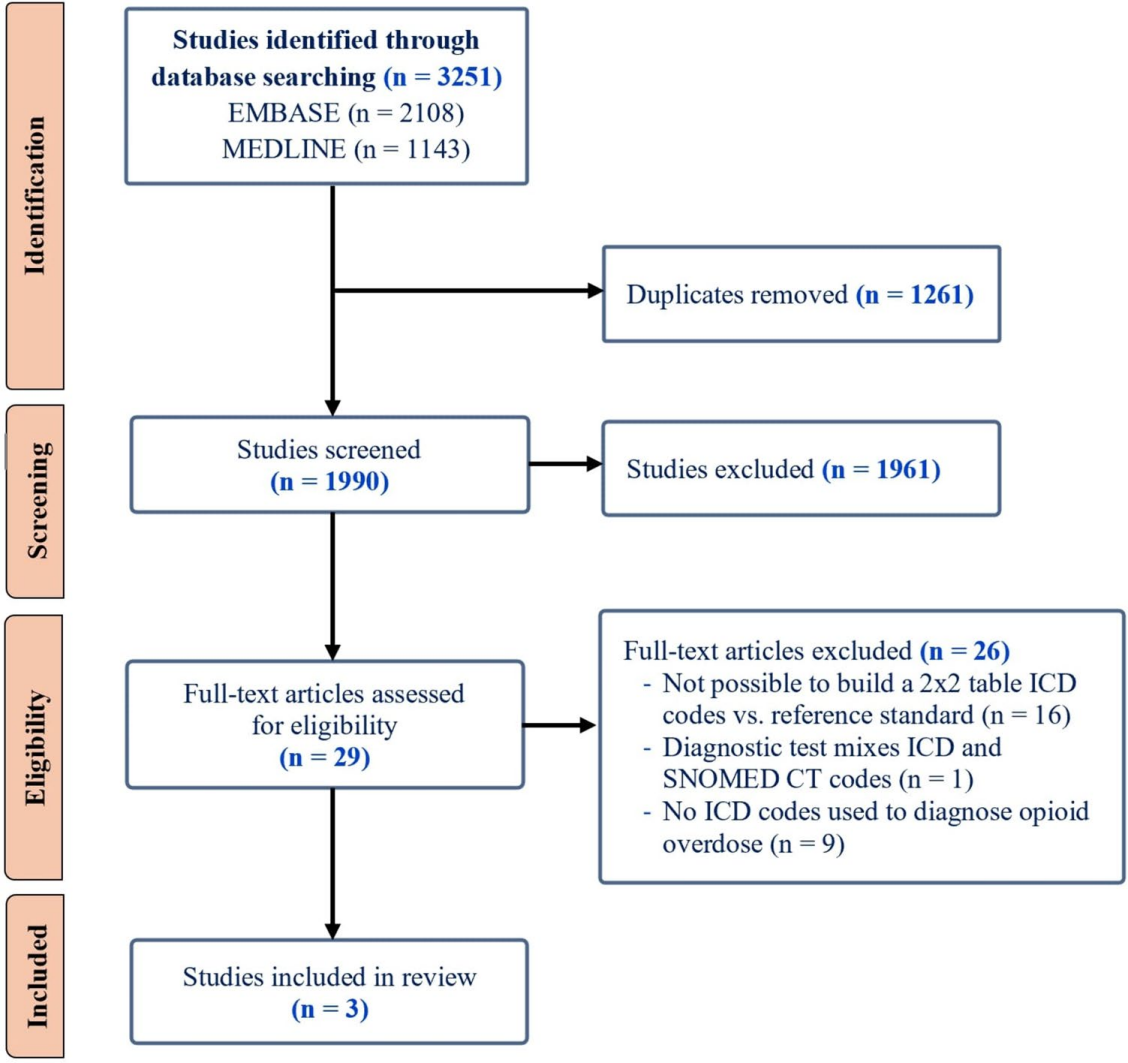
PRISMA flow diagram illustrating selection process of relevant articles. ICD, International Classification of Diseases; SNOMED CT, Systematized Nomenclature of Medicine Clinical Terms

Figure 1 presents the PRISMA flowchart of study selection process. We identified a total of 1990 unique studies through database search, of which three articles were included upon completion of the selection process. No studies were selected through snowball manual search.

### Characteristics of included studies

Characteristics of the three included studies are summarized in Table 1. The first study (Rowe et al., 2017) was conducted in the United States and used data from 3203 emergency department visits between 2012 and 2014 made by 804 patients (mean age 56 years, 58.1% male) who were prescribed at least daily opioids for at least 3 consecutive months. The outcome was OOD events, assessed using several ICD-9 algorithms as index tests, and the medical chart review, conducted by four research staff, including one physician and one nurse practitioner, as the reference standard.

**Table 1:** Characteristics of the three studies included in the review

Author, year	Country; years evaluated	Study design; data source	Study population	Sample size	Mean age (years)	% male	Outcome of interest	ICD Revision algorithms used to classify participants^a^	Reference standard (RS)
Rowe et al., 2017	USA; 2012–2014	Cross-sectional; data on emergency department visits abstracted from electronic medical records for patients prescribed long-term opioids for pain from six safety net primary care clinics in San Francisco, California	Patients at six selected clinics who were prescribed at least daily opioids by their primary care provider for at least 3 consecutive months, and visited the safety net emergency department from January 1, 2012, to December 31, 2014	*n* = 3203 visits (804 unique people)	56.0	58.1	Opioid overdose events	ICD-9 codes (six primary algorithms)^b^: *CV1*: E850.0; E850.1; E850.2; 965.01; 965.02; 965.09 *CV2*: CV1 codes; 977.9; E858.9; E980.0 *CV3*: CV2 codes; 965 *CV4*: CV3 codes; 305.5 *CV5*: CV4 codes; 305.9; 305.93 *CV6*: CV5 codes; 305	RS: Medical chart review was conducted by four research staff, including one physician and one nurse practitioner. At least 62.5% of the eligible charts were assessed by two or more reviewers. Agreement between reviewers was not reported Case definition: Opioid overdose events were defined as events in which the documenting physician determined that a patient was unarousable or not breathing sufficiently due to opioids
Green et al., 2019	USA; 2008–2014	Cross-sectional; administrative, clinical, inpatient, outpatient, and claims data from Kaiser Permanente Northwest in Oregon and southwest Washington State (development and validation datasets), and clinical care information from non‐Kaiser Permanente Northwest settings	Development and validation samples included members of Kaiser Permanente Northwest suspected with overdoses or at risk for overdose from January 1, 2008, to December 31, 2014	Development dataset: *n* = 872 events (845 unique people)	48.8	39.5	Opioid overdose events	ICD-9 and ICD-10 (Initial algorithm)^c^ *ICD-9 codes*: poisoning by opium (alkaloids) unspecified (965.00); poisoning by heroin (965.01); poisoning by methadone (965.02); poisoning by other opiates and related narcotics (965.09); accidental poisoning by heroin (E850.0); accidental poisoning by methadone (E850.1); accidental poisoning by other opiates and related narcotics (E850.2); cause of death: poisoning by opiates and related narcotics (9650) *ICD-10 codes*: cause of death: poisoning by opium (T40.0); poisoning by heroin (T40.1); poisoning by other opioids (T40.2); poisoning by methadone (T40.3); poisoning by other synthetic narcotic (T40.4); accidental poisoning by and exposure to narcotics and psychodysleptics, not elsewhere classified (X42); intentional self‐poisoning by and exposure to narcotics and psychodysleptics, not elsewhere classified (X62); undetermined poisoning by and exposure to narcotics and psychodysleptics, not elsewhere classified (Y12)	RS: Medical chart review was conducted by trained professional chart auditors. Two reviewers independently assessed 100% of charts. Agreement between reviewers was not reported Case definition: Opioid overdose events were defined using several criteria, including a qualified clinician’s indication that an urgent medical event involved an overdose or a suicide attempt involving medications/drugs, the finding of symptoms suggestive of overdose, rescue efforts with partial or full response to administration of naloxone/Narcan, the clinician's indication or clear evidence that substance-involved overdose was opioid-related^d^
				Validation dataset: *n* = 1136 events (1100 unique people)	46.9	39.6			
Gladstone et al., 2016	Canada; 2003–2010	Cross-sectional; mortality data abstracted from the Chief Coroner and Statistics Canada Vital Statistics Databases for Ontario province	All drug- or alcohol-related deaths involving prescription opioids among Ontario residents that occurred within their province between January 1, 2003, and December 31, 2010	*n* = 88,500	N/R	N/R	Prescription opioid–related deaths	ICD-10 codes (five algorithms): *Algorithm 1*: Underlying cause of death: poisoning (X40–X49, X60–X69, X85–X90, Y10–Y19) and multiple cause of death: prescription opioid poisoning (T402, T403, T404) *Algorithm 2*: Underlying cause of death: poisoning (X40–X49, X60–X69, X85–X90, Y10–Y19) and multiple cause of death: prescription opioid poisoning and/or other unspecified narcotics (T402, T403, T404, T406) *Algorithm 3*: Multiple cause of death: prescription opioid poisoning (T402, T403, T404) *Algorithm 4*: Multiple cause of death: prescription opioid poisoning or other unspecified narcotics (T402, T403, T404, T406) *Algorithm 5*: Underlying cause of death or multiple cause of death: narcotic poisoning (X42, X62, Y12, T509) and not multiple cause of death: illicit opioids (T401, T405, T407–T409) or multiple cause of death: prescription opioid poisoning or other unspecified narcotics (T402, T403, T404, T406)	RS: Coroner’s report review was independently conducted by two abstractors. Both extractors agreed 100% about the causes of death for a sample of the charts Case definition: Prescription opioid-related deaths were defined as cases where the coroner’s report indicated either that prescription opioid concentrations were present at high enough levels to cause death or that a combination of drugs caused death that included one or more prescription opioids detected at a clinically significant concentration

*ICD*, International Classification of Diseases; *RS*, reference standard; *CV*, composite variable; *N/R*, not reported

^a ^The presence of at least one code in the list classified a visit as positive for opioid overdose (OOD) or prescription opioid–related deaths (POD). Visits without any of these codes mentioned were classified as negative for OOD or POD

^b^ Additional composite variables referred to as clinically related exploratory composite variables (CV7–CV13) and clinically unrelated exploratory composite variables (CV14–CV18) are not presented

^c^ Other specific ICD algorithms not presented were used to classify opioid overdose as heroin involvement, suicide/suicide attempt, and abuse involvement

^d ^See the Data S2 Supplement B file in Green et al. (2019) for more details

The second study (Green et al., 2019) included administrative, clinical, inpatient, outpatient, and claims information on 872 (development dataset; mean age 48.8 years, 39.5% male) and 1136 (validation dataset; mean age 46.9 years, 39.6% male) events experienced by 845 and 1100 members of Kaiser Permanente Northwest in Oregon and southwest Washington State, who were suspected of or at risk of OOD from 2008 to 2014, respectively. Clinical care information from non‐Kaiser Permanente Northwest settings was also considered. As in the previous study, the outcome was OOD events, and the reference standard was medical chart review which was independently conducted by two trained professionals for 100% of eligible charts. The initial index test algorithms included a combination of ICD-9 codes for non-fatal and fatal events and ICD-10 codes for fatal events.

The last study (Gladstone et al., 2016) was conducted in Canada and handled the data of 88,550 drug- or alcohol-related death cases among Ontario residents between 2003 and 2010. POD were the outcome, identified using five ICD-10 algorithms from Statistics Canada Vital Statistics Databases for Ontario, as index tests, and CRR, conducted by two independent extractors from the Chief Coroner Database for Ontario, as the reference standard.

### Accuracy of ICD algorithms in identifying OOD

Table 2 presents the estimates of sensitivity and specificity of ICD algorithms assuming that the reference standard is perfect, as well as the 2 × 2 table contents of agreement between the two tests, and the outcome prevalence based on the reference standard in the eligible studies. The three studies varied in the ICD algorithms, the version of ICD (9 and 10), and the reference standards.

**Table 2:** Estimates (95% confidence interval) of sensitivity and specificity and agreement of ICD algorithms (index test) versus reference standard (*RS*), and prevalence of opioid overdose–related events in the three studies included in the review

Author, year	Accuracy of ICD algorithm vs. RS				2 × 2 table of agreement between ICD algorithm and RS				Prevalence of the outcome based on the RS (%)
	ICD Revision	ICD algorithm^a^	Sensitivity	Specificity	True positives	False negatives	False positives	True negatives	
Rowe et al., 2017^b^	ICD-9	CV1	0.250 (0.136–0.378)	0.999 (0.998–1.000)	11	33	2	3157	1.37
		CV2	0.364 (0.220–0.500)	0.998 (0.996–0.999)	16	28	7	3152	1.37
		CV3	0.386 (0.242–0.533)	0.998 (0.996–0.999)	17	27	7	3152	1.37
		CV4	0.432 (0.286–0.588)	0.994 (0.991–0.996)	19	25	20	3139	1.37
		CV5	0.500 (0.373–0.679)	0.982 (0.977–0.987)	22	22	57	3102	1.37
		CV6	0.568 (0.436–0.727)	0.962 (0.948–0.972)	25	19	119	3040	1.37
Green et al., 2019	ICD-9 and ICD-10	Initial algorithm (Development dataset)	0.979 (0.960–0.990)	0.889 (0.856–0.916)	414	9	50	399	48.50
		Initial algorithm (Validation dataset)	0.972 (0.955–0.984)	0.846 (0.813–0.875)	588	17	82	449	53.26
Gladstone et al., 2016	ICD-10	Algorithm 1	0.72 (0.68–0.75)	0.99 (0.99–0.99)	388	154	37	87,921	0.61
		Algorithm 2	0.75 (0.71–0.78)	0.99 (0.99–0.99)	404	138	43	87,915	0.61
		Algorithm 3	0.72 (0.68–0.76)	0.99 (0.99–0.99)	390	152	47	87,911	0.61
		Algorithm 4	0.75 (0.71–0.79)	0.99 (0.99–0.99)	407	135	59	87,899	0.61
		Algorithm 5	0.89 (0.87–0.92)	0.99 (0.99–0.99)	485	57	207	87,751	0.61

ICD, International Classification of Diseases; RS, reference standard; CV, composite variable (refers to algorithms used)

^a^ See Table 1 for details of specific ICD codes for each algorithm

^b^ Adding ICD-9 codes to CVs increased sensitivity to 100% (92–100%) and lowered specificity to 81% (78.9–83.2%) for CV18

Compared with medical chart review (Rowe et al., 2017), the six primary ICD-9 algorithms showed very low sensitivity ranging from 25% (95% confidence interval 13.6–37.8) for more precise opioid-poisoning ICD-9 code algorithm to 56.8% (43.6–72.7) when expanding the initial ICD-9 algorithm to include non-specified and general drug poisoning and drug abuse codes. The corresponding specificity estimate was highest with the more targeted ICD-9 algorithm (99.9%) but reduced to 96.2% with the inclusion of less specific codes. The prevalence of OOD events estimated based on the reference standard was very low (1.37%).

Green et al. (2019) also used medical chart review as the reference standard but in a population where the OOD event prevalence was very high (48.5–53.26%) based on the reference standard. The combination of ICD-9 and ICD-10 codes for poisoning by opioids and related narcotics as an index test showed improved sensitivity over 97% in diagnosing OOD events, whereas the specificity values were 88.9% (85.6–91.6) and 84.6% (81.3–87.5) in development and validation datasets, respectively.

Finally, the work by Gladstone et al. (2016) reported moderate to good sensitivity of ICD-10 algorithms ranging from 72% (68–75%) to 89% (87–92%) and consistently excellent specificity of 99% in identifying POD, assuming CRR is a gold standard. The prevalence of POD detected by CRR was rare (0.61%).

### Study quality assessment

Table 3 shows the assessment of bias and applicability of eligible studies. No applicability concerns were identified for all three included studies. The risk of bias was judged to be “low” in all four QUADAS-2 domains in one study (Gladstone et al., 2016), and only in the patient selection and the index test domains for the other two studies (Green et al., 2019; Rowe et al., 2017). In Rowe et al. (2017), given that the medical chart review was applied by more than one person and that there was no information on inter-reader agreement, it was unclear whether the reference standard was likely to correctly classify OOD events, or whether all patients received the same reference standard, thus justifying the “unclear” score assigned for bias assessment in both reference standard and flow and time domains. In Green et al. (2019), bias was scored as “high” in the reference standard domain, as Kaiser Permanente Northwest chart auditors were provided with inclusion diagnoses derived from results of the code-based algorithm, suggesting that the reference standard results were interpreted with knowledge of the results of the index test. As in Rowe et al. (2017), the study by Green et al. (2019) did not provide information on inter-reader agreement for the reference standard, thus justifying the “unclear” score assigned to the bias assessment for the flow and time domain.

**Table 3:**
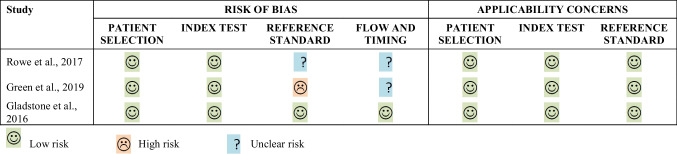
Assessment of bias and applicability for eligible studies using QUADAS-2 tool

### Estimation of validity of ICD-10 codes and CRR in diagnosing POD

Table 4 reports estimates of sensitivity and specificity of each of the five ICD-10 code algorithms and CRR in diagnosing POD derived from BLCM. The sensitivity estimates of the two tests did not substantially vary according to the prior values used, but rather according to the ICD-10 algorithms. The median values (95% BCI) estimated for the sensitivity of ICD-10 algorithms 1 to 4 were similar, ranged between 84.4% (71.0–99.2) and 87.1% (75.8–99.3), while that of ICD-10 algorithm 5 was higher (94.5% (88.6–99.7)). As for CRR, the sensitivity estimates were similar when CRR was compared to ICD-10 algorithms 1 to 4 and ranged between 93.3% (86.3–99.7) and 95.4% (90.1–99.8) but were lower in the analysis comparing CRR to ICD-10 algorithm 5, ranging between 83.6% (69.8–99.1) when the priors were the least informative to 86.6% (75.5–99.3) when they were the most informative. The sensitivity estimates of both tests were imprecise due to the scarcity of the outcome. Conversely, the median estimates for specificity of both tests were similar, near-perfect (median values ≥ 99.8%), and highly precise (minimum 95% BCI lower limit of 99.8%).

**Table 4:** Posterior medians and 95% Bayesian credible intervals of sensitivity and specificity of ICD-10 algorithms and coroner’s report review (CRR) in diagnosing prescription opioid–related deaths using Bayesian latent class models

	Se ICD	Sp ICD	Se CRR	Sp CRR
Scenario 1^a^				
ICD-10 algorithm 1	0.866 (0.756–0.993)	1.000 (1.000–1.000)	0.954 (0.901–0.998)	0.999 (0.998–1.000)
ICD-10 algorithm 2	0.871 (0.758–0.993)	1.000 (0.999–1.000)	0.950 (0.893–0.997)	0.999 (0.998–1.000)
ICD-10 algorithm 3	0.866 (0.756–0.993)	1.000 (0.999–1.000)	0.944 (0.881–0.997)	0.999 (0.998–1.000)
ICD-10 algorithm 4	0.871 (0.759–0.993)	1.000 (0.999–1.000)	0.933 (0.863–0.997)	0.999 (0.998–1.000)
ICD-10 algorithm 5	0.945 (0.886–0.997)	0.998 (0.998–1.000)	0.866 (0.755–0.993)	1.000 (0.999–1.000)
Scenario 2^b^				
ICD-10 algorithm 1	0.844 (0.710–0.992)	1.000 (1.000–1.000)	0.954 (0.901–0.998)	0.999 (0.998–1.000)
ICD-10 algorithm 2	0.863 (0.739–0.993)	1.000 (0.999–1.000)	0.950 (0.893–0.997)	0.999 (0.998–1.000)
ICD-10 algorithm 3	0.847 (0.714–0.992)	1.000 (0.999–1.000)	0.944 (0.881–0.997)	0.999 (0.998–1.000)
ICD-10 algorithm 4	0.866 (0.745–0.993)	1.000 (0.999–1.000)	0.934 (0.863–0.997)	0.999 (0.998–1.000)
ICD-10 algorithm 5	0.945 (0.886–0.997)	0.999 (0.998–1.000)	0.836 (0.698–0.991)	1.000 (0.999–1.000)

Prior uniform (0.00–0.102) was used for prevalence

*ICD*, International Classification of Diseases; *CRR*, coroner’s report review; *Se*, sensitivity; *Sp*, specificity

^a^ Scenario 1 used uniform prior distributions of (0.75–1.00) for the sensitivity of the CRR and of the ICD algorithm, and (0.90–1.00) for the specificity of the CRR and of the ICD algorithm

^b ^Scenario 2 used uniform prior distributions of (0.00–1.00) for the sensitivity of the CRR and of the ICD algorithm, and (0.00–1.00) for the specificity of the CRR and of the ICD algorithm

## Discussion

To our knowledge, this study is the first to systematically review published articles assessing the validity of ICD codes for OOD events and is the first to estimate the sensitivity and specificity of ICD codes in the absence of a gold standard for identifying POD. Our review demonstrated that very few studies have focused on examining the validity of ICD codes as diagnostic tools for OOD-related events, as previously observed for illicit drugs (McGrew et al., 2020). Included studies were widely heterogeneous with regard to study population, data sources, ICD Revision (9th and/or 10th) and ICD algorithms, specific OOD events considered, and reference standards. This explains the variation in the sensitivity and specificity and calls for cautious interpretation of estimates of the frequency of OOD based on ICD codes in administrative data which must be done with respect to the specific context of each included study.

Despite the heterogeneity among the eligible studies, one common point highlighted is the good specificity of ICD algorithms, suggesting that ICD codes may be a useful tool for documenting the absence of OOD cases in the administrative data, at least when the outcome is relatively common. Conversely, the sensitivity of ICD codes to correctly detect cases of OOD varied widely between included studies, and between ICD algorithms within a study. Indeed, when used alone, ICD-9 algorithms proved poorly sensitive in detecting OOD events, but sensitivity increased with the addition of codes in the algorithms at the expense of a decline in specificity (Rowe et al., 2017). Compared to ICD-9, ICD-10 algorithms alone showed moderate to good sensitivity (Gladstone et al., 2016), but the ICD-9 and ICD-10 sensitivity estimates reported in these two studies are uncertain because the prevalence of OOD events in both study populations was uncommon. In contrast, the combination of ICD-9 and ICD-10 (Green et al., 2019) showed better and precise sensitivity estimates, but the study population included was one already under surveillance for addiction, and the presumed true prevalence of OOD events was very high, at about 50%.

Results from the studies by Rowe et al. (2017) and Green et al. (2019), despite requiring cautious interpretation due to suspected risk of bias, highlight the challenge of getting precise sensitivity estimates of ICD codes at the population level. A precise estimate of ICD code sensitivity requires a context where the prevalence of outcome is relatively frequent, but in this context, it is likely that the search for OOD will be more extensive than in a less at-risk population. Conducting a study in a more general population would require a very large sample size to obtain a sufficient number of positive tests, which may not be feasible. In addition, the improvement in sensitivity estimates generally came at the cost of decreased specificity estimates, leading to an overestimation of prevalence estimates using ICD codes. However, the good sensitivity and excellent specificity estimates of algorithm 5, when CRR is assumed perfect (Gladstone et al., 2016), would result in less bias in estimating the prevalence of POD than the other algorithms and studies in a range of contexts in terms of the frequency of OOD-related events.

This makes BLCM crucial to estimate the performance of ICD codes in the absence of a gold standard, and ultimately adjust for misclassification error in estimating the frequency of OOD-related events. Our analyses using BLCM allowed us to estimate the sensitivity and specificity of ICD-10 algorithms and CRR in diagnosing POD in the absence of a perfect test. Estimates from our models showed that CRR was not a perfect diagnostic test for POD. Regardless of prior values, the ICD-10 algorithm specificity estimates from our models were similar to those reported by the authors assuming that the CRR is perfect. Given the high relative weight of negative cases (which accounts for over 99% of the 88,500 death cases) and the use of uniform priors in our models, the estimation of the posterior distribution for specificity was largely driven by the observed data.

As observed for specificity, our models showed that the estimated sensitivity values of the ICD-10 algorithms remained substantially consistent regardless of the prior values assigned. In contrast, the ICD-10 algorithm sensitivity estimates reported by the authors systematically underestimated the sensitivity estimates of our models. This result demonstrates the relevance of controlling for misclassification error when evaluating the validity of imperfect diagnostic tests and may support the use of ICD-10 codes in detecting POD. The variability in the sensitivity estimates across ICD-10 algorithms suggests the need for standardization of OOD case definitions, which will improve the quality and comparability of data on OOD occurrence estimates across the country and over time for better service planning and research (Vivolo-Kantor et al., 2021). It is also essential that more studies evaluate ICD codes for OOD to obtain better prior values on certain ICD algorithms, and that public health estimation of OOD prevalence uses such available priors to adjust for ICD algorithm misclassification bias.

### Strengths and limitations

The strengths of this study include the conduct of a comprehensive review of published studies on the validity of ICD codes for OOD, and rigorous assessment of the quality of eligible studies using the QUADAS-2 tool. Our review is the first study to use BLCM for estimating the misclassification-adjusted sensitivity and specificity of both ICD-10 codes and CRR in identifying POD in Canada. Our estimates of the sensitivity and specificity of ICD-10 codes and CRR can therefore be used as priors in other studies, whether in Canada or in other countries where the context of our study could be generalized, although the information on sensitivity estimates remains uncertain. Due to the lack of prior information on the sensitivity and specificity of both ICD-10 codes and CRR in diagnosing POD, we considered several scenarios of uniform prior distributions, providing greater insight into plausible variations in test sensitivity and specificity estimates as a function of the prior values used.

Several limitations can be highlighted. The three included studies were based on specific populations with higher risk of OOD than what would be expected in the general population, including people under routine medical care, whether for long-term opioid use for pain management (Rowe et al., 2017) or for comprehensive inpatient and outpatient medical care including addiction and mental health treatment (Green et al., 2019), as well as alcohol- or drug-related deaths that have undergone forensic evaluation by the coroner (Gladstone et al., 2016). The data reported in these studies may differ from national data on OOD which would include OOD events occurring in any individual, limiting the generalizability of the sensitivity and specificity estimates of ICD codes reported in these studies to the general population, and prompting caution in their interpretation. We reported a high or unclear risk of bias in two QUADAS domains in two of the three studies included in the review, also urging caution in interpreting the reported ICD code validity estimates. In addition, we were unable to perform a meta-analysis to estimate summary measures of ICD code sensitivity and specificity due to the small number of eligible studies and the significant heterogeneity observed in these studies, as mentioned above. Finally, the sensitivity estimates of the ICD-10 algorithms and CRR in the absence of a gold standard are imprecise due to the very low prevalence of POD (Gladstone et al., 2016) and the use of uniform prior distributions for all of the parameters.

## Conclusion

As the opioid crisis expands across Canada and around the world, the ICD coding system is increasingly emerging as an essential tool for tracking and monitoring opioid overdose events in administrative data from emergency departments, emergency services, hospitalizations, outpatient visits, or claims. Our review highlighted the paucity of studies on the validity of ICD codes in the diagnosis of OOD events. Estimates of the misclassification-adjusted sensitivity and specificity of ICD codes support the usefulness of relying on ICD codes as diagnostic tools for identifying POD in Canada and provide prior information to better assess the validity of ICD codes for OOD in similar populations.

## Contributions to knowledge

What does this study add to existing knowledge?

- Evidence on the validity of ICD codes for opioid overdose (OOD) is rare, with none available for classifying the issue in the general population.
- ICD-10 algorithm sensitivity estimates in classifying prescription opioid–related deaths (POD) in the absence of a gold standard are moderate and would result in the omission of some truly positive individuals.
- Moderate sensitivity and excellent specificity estimates of ICD-10 algorithms would result in only minimal bias in estimating the prevalence of POD in a situation where this event is rare, whereas this impact could be different in populations where the use of opioid is more frequent.

What are the key implications for public health interventions, practice, or policy?

- Public health programs responding to the opioid crisis in Canada can rely on ICD-10 algorithms as diagnostic tools for POD and should encourage more studies examining the validity of ICD codes for OOD to be conducted in various population strata, in order to adjust for misclassification error in estimating population-level frequency estimates.

## Supplementary Information

Below is the link to the electronic supplementary material.

Supplementary file1 (DOCX 27 KB)

Supplementary file2 (DOCX 30 KB)

Supplementary file3 (DOCX 31 KB)

Supplementary file4 (DOCX 22 KB)

## Data Availability

The data used in this study can be found in Tables 1, 2, 3, and 4.

## Code availability

The base code is presented in Online Resource 4.

## References

[CR1] Algera, M. H., Olofsen, E., Moss, L., Dobbins, R. L., Niesters, M., van Velzen, M., Groeneveld, G. J., Heuberger, J., Laffont, C. M., & Dahan, A. (2021). Tolerance to opioid-induced respiratory depression in chronic high-dose opioid users: A model-based comparison with opioid-naïve individuals. *Clinical Pharmacology & Therapeutics,**109*(3), 637–645. 10.1002/cpt.2027 32865832 10.1002/cpt.2027 PMC7983936

[CR2] Anastasiou, C., & Yazdany, J. (2022). Review of publications evaluating opioid use in patients with inflammatory rheumatic disease. *Current Opinion in Rheumatology,**34*(2), 95–102. 10.1097/bor.0000000000000868 35044328 10.1097/BOR.0000000000000868 PMC8974237

[CR3] Angelidou, E., Kostoulas, P., & Leontides, L. (2014). Bayesian validation of a serum and milk ELISA for antibodies against Mycobacterium avium subspecies paratuberculosis in Greek dairy goats across lactation. *Journal of Dairy Science,**97*(2), 819–828. 10.3168/jds.2013-7218 24359824 10.3168/jds.2013-7218

[CR4] Arango-Sabogal, J. C., Fecteau, G., Paré, J., Roy, J. P., Labrecque, O., Côté, G., Wellemans, V., Schiller, I., Dendukuri, N., & Buczinski, S. (2018). Estimating diagnostic accuracy of fecal culture in liquid media for the detection of Mycobacterium avium subsp. paratuberculosis infections in Québec dairy cows: A latent class model. *Preventive Veterinary Medicine,**160*, 26–34. 10.1016/j.prevetmed.2018.09.025 10.1016/j.prevetmed.2018.09.025 30388995

[CR5] Bateman, J. T., Saunders, S. E., & Levitt, E. S. (2023). Understanding and countering opioid-induced respiratory depression. *Br J Pharmacol,**180*(7), 813–828. 10.1111/bph.15580 34089181 10.1111/bph.15580 PMC8997313

[CR6] Berman, J., Francoz, D., Dufour, S., & Buczinski, S. (2019). Bayesian estimation of sensitivity and specificity of systematic thoracic ultrasound exam for diagnosis of bovine respiratory disease in pre-weaned calves. *Preventive Veterinary Medicine,**162*, 38–45. 10.1016/j.prevetmed.2018.10.025 10.1016/j.prevetmed.2018.10.025 30621897

[CR7] Branscum, A. J., Gardner, I. A., & Johnson, W. O. (2005). Estimation of diagnostic-test sensitivity and specificity through Bayesian modeling. *Preventive Veterinary Medicine,**68*(2–4), 145–163. 10.1016/j.prevetmed.2004.12.005 15820113 10.1016/j.prevetmed.2004.12.005

[CR8] Chartash, D., Paek, H., Dziura, J. D., Ross, B. K., Nogee, D. P., Boccio, E., Hines, C., Schott, A. M., Jeffery, M. M., Patel, M. D., Platts-Mills, T. F., Ahmed, O., Brandt, C., Couturier, K., & Melnick, E. (2019). Identifying opioid use disorder in the emergency department: Multi-system electronic health record-based computable phenotype derivation and validation study. *JMIR Medical Informatics,**7*(4), e15794. 10.2196/15794 31674913 10.2196/15794 PMC6913746

[CR9] Cheung, A., Dufour, S., Jones, G., Kostoulas, P., Stevenson, M. A., Singanallur, N. B., & Firestone, S. M. (2021). Bayesian latent class analysis when the reference test is imperfect. *Revue Scientifique et Technique de l’OIE,**40*(1), 271–286. 10.20506/rst.40.1.3224 34140724 10.20506/rst.40.1.3224

[CR10] Coben, J. H., Davis, S. M., Furbee, P. M., Sikora, R. D., Tillotson, R. D., & Bossarte, R. M. (2010). Hospitalizations for poisoning by prescription opioids, sedatives, and tranquilizers. *American Journal of Preventive Medicine,**38*(5), 517–524. 10.1016/j.amepre.2010.01.022 20409500 10.1016/j.amepre.2010.01.022

[CR11] Collins, J., & Huynh, M. (2014). Estimation of diagnostic test accuracy without full verification: A review of latent class methods. *Statistics in Medicine,**33*(24), 4141–4169. 10.1002/sim.6218 24910172 10.1002/sim.6218 PMC4199084

[CR12] Dalal, S., & Bruera, E. (2019). Pain management for patients with advanced cancer in the opioid epidemic era. *American Society of Clinical Oncology Educational Book,**39*, 24–35. 10.1200/edbk_100020 10.1200/EDBK_100020 31099619

[CR13] Fiore, J. F., Jr., El-Kefraoui, C., Chay, M. A., Nguyen-Powanda, P., Do, U., Olleik, G., Rajabiyazdi, F., Kouyoumdjian, A., Derksen, A., Landry, T., Amar-Zifkin, A., Bergeron, A., Ramanakumar, A. V., Martel, M., Lee, L., Baldini, G., & Feldman, L. S. (2022). Opioid versus opioid-free analgesia after surgical discharge: A systematic review and meta-analysis of randomised trials. *Lancet,**399*(10343), 2280–2293. 10.1016/s0140-6736(22)00582-7 35717988 10.1016/S0140-6736(22)00582-7

[CR14] Gelman, A., Meng, X.-L., & Stern, H. (1996). Posterior predictive assessment of model fitness via realized discrepancies. *Statistica Sinica, 6*(4), 733–760. http://www.jstor.org/stable/24306036. Accessed 15 Jan 2014.

[CR15] Gladstone, E., Smolina, K., Morgan, S. G., Fernandes, K. A., Martins, D., & Gomes, T. (2016). Sensitivity and specificity of administrative mortality data for identifying prescription opioid-related deaths [Research Support, Non-U.S. Gov't]. *Canadian Medical Association Journal, 188*(4), E67–E72. 10.1503/cmaj.150349 10.1503/cmaj.150349 PMC4771548 26622006

[CR16] Green, C. A., Perrin, N. A., Janoff, S. L., Campbell, C. I., Chilcoat, H. D., & Coplan, P. M. (2017). Assessing the accuracy of opioid overdose and poisoning codes in diagnostic information from electronic health records, claims data, and death records. *Pharmacoepidemiol Drug Saf,**26*(5), 509–517. 10.1002/pds.4157 28074520 10.1002/pds.4157

[CR17] Green, C. A., Perrin, N. A., Hazlehurst, B., Janoff, S. L., DeVeaugh-Geiss, A., Carrell, D. S., Grijalva, C. G., Liang, C., Enger, C. L., & Coplan, P. M. (2019). Identifying and classifying opioid-related overdoses: A validation study. *Pharmacoepidemiology and Drug Safety,**28*(8), 1127–1137. 10.1002/pds.4772 31020755 10.1002/pds.4772 PMC6767606

[CR18] Hallgren, K. A., Witwer, E., West, I., Baldwin, L. M., Donovan, D., Stuvek, B., Keppel, G. A., Mollis, B., & Stephens, K. A. (2020). Prevalence of documented alcohol and opioid use disorder diagnoses and treatments in a regional primary care practice-based research network. *Journal of Substance Abuse Treatment,**110*, 18–27. 10.1016/j.jsat.2019.11.008 10.1016/j.jsat.2019.11.008 PMC7255441 31952624

[CR19] Kelsall, D., & Bowes, M. J. (2016). No standards: Medicolegal investigation of deaths. *Canadian Medical Association Journal,**188*(3), 169. 10.1503/cmaj.160041 26833736 10.1503/cmaj.160041 PMC4754172

[CR20] Khanna, A. K., Bergese, S. D., Jungquist, C. R., Morimatsu, H., Uezono, S., Lee, S., Ti, L. K., Urman, R. D., McIntyre, R., Jr., Tornero, C., Dahan, A., Saager, L., Weingarten, T. N., Wittmann, M., Auckley, D., Brazzi, L., Le Guen, M., Soto, R., Schramm, F., … Overdyk, F. J. (2020). Prediction of opioid-induced respiratory depression on inpatient wards using continuous capnography and oximetry: An international prospective, observational trial. *Anesthesia & Analgesia,**131*(4), 1012–1024. 10.1213/ane.0000000000004788 32925318 10.1213/ANE.0000000000004788 PMC7467153

[CR21] Kostoulas, P., Nielsen, S. S., Branscum, A. J., Johnson, W. O., Dendukuri, N., Dhand, N. K., Toft, N., & Gardner, I. A. (2017). STARD-BLCM: Standards for the reporting of diagnostic accuracy studies that use Bayesian Latent Class Models. *Preventive Veterinary Medicine,**138*, 37–47. 10.1016/j.prevetmed.2017.01.006 10.1016/j.prevetmed.2017.01.006 28237234

[CR22] Lahti, R. A., Korpi, H., & Vuori, E. (2009). Blood-positive illicit-drug findings: Implications for cause-of-death certification, classification and coding. *Forensic Science International,**187*(1–3), 14–18. 10.1016/j.forsciint.2009.02.007 19303228 10.1016/j.forsciint.2009.02.007

[CR23] Liang, S. Y., Phillips, K. A., Wang, G., Keohane, C., Armstrong, J., Morris, W. M., & Haas, J. S. (2011). Tradeoffs of using administrative claims and medical records to identify the use of personalized medicine for patients with breast cancer. *Medical Care,**49*(6), e1-8. 10.1097/MLR.0b013e318207e87e 21422962 10.1097/MLR.0b013e318207e87e PMC3383782

[CR24] Lutomski, J., Byrne, B., Devane, D., & Greene, R. (2012). Increasing trends in atonic postpartum haemorrhage in Ireland: An 11-year population-based cohort study. *119*(3), 306–314. 10.1111/j.1471-0528.2011.03198.x 10.1111/j.1471-0528.2011.03198.x 22168794

[CR25] Madadi, P., Hildebrandt, D., Lauwers, A. E., & Koren, G. (2013). Characteristics of opioid-users whose death was related to opioid-toxicity: A population-based study in Ontario, Canada. *Plos ONE,**8*(4), e60600. 10.1371/journal.pone.0060600 23577131 10.1371/journal.pone.0060600 PMC3618438

[CR26] McGrew, K. M., Homco, J. B., Garwe, T., Dao, H. D., Williams, M. B., Drevets, D. A., Jafarzadeh, S. R., Zhao, Y. D., & Carabin, H. (2020). Validity of International Classification of Diseases codes in identifying illicit drug use target conditions using medical record data as a reference standard: A systematic review. *Drug Alcohol Depend,**208*, 107825. 10.1016/j.drugalcdep.2019.107825 31982637 10.1016/j.drugalcdep.2019.107825 PMC9533471

[CR27] McLean, M. (2017). Contradictory coroners? Decision-making in death investigations. *Journal of Clinical Pathology,**70*(9), 787–791. 10.1136/jclinpath-2017-204333 28396386 10.1136/jclinpath-2017-204333

[CR28] Moher, D., Liberati, A., Tetzlaff, J., & Altman, D. G. (2009). Preferred reporting items for systematic reviews and meta-analyses: The PRISMA statement. *BMJ, 339*, b2535. 10.1136/bmj.b2535 PMC3090117 21603045

[CR29] Ogeil, R. P., Dwyer, J., Bugeja, L., Heilbronn, C., Lubman, D. I., & Lloyd, B. (2018). Pharmaceutical opioid overdose deaths and the presence of witnesses. *International Journal of Drug Policy,**55*, 8–13. 10.1016/j.drugpo.2017.12.020 10.1016/j.drugpo.2017.12.020 29433040

[CR30] Parai, J. L., Kreiger, N., Tomlinson, G., & Adlaf, E. M. (2006). The validity of the certification of manner of death by Ontario coroners. *Annals of Epidemiology,**16*(11), 805–811. 10.1016/j.annepidem.2006.01.006 16621598 10.1016/j.annepidem.2006.01.006

[CR31] Parthvi, R., Agrawal, A., Khanijo, S., Tsegaye, A., & Talwar, A. (2019). Acute opiate overdose: An update on management strategies in emergency department and critical care unit. *26*(3), e380–e387.10.1097/mjt.0000000000000681 10.1097/MJT.0000000000000681 28952972

[CR32] Penington Institute. (2022). *Global Overdose Snapshot 2022*. Penington Institute. Retrieved 2024 Jan 7 from https://www.penington.org.au/wp-content/uploads/2022/09/Penington-Institute-Global-Overdose-Snapshot-2022.pdf

[CR33] PHAC. (2023). *Opioid- and stimulant-related harms in Canada [Internet]*. Public Health Agency of Canada. Retrieved 2024 Jan 8 from https://health-infobase.canada.ca/substance-related-harms/opioids-stimulants/

[CR34] Ranapurwala, S. I., Alam, I. Z., Pence, B. W., Carey, T. S., Christensen, S., Clark, M., Chelminski, P. R., Wu, L. T., Greenblatt, L. H., Korte, J. E., Wolfson, M., Douglas, H. E., Bowlby, L. A., Capata, M., & Marshall, S. W. (2023). Development and validation of an electronic health records-based opioid use disorder algorithm by expert clinical adjudication among patients with prescribed opioids. *Pharmacoepidemiology and Drug Safety,**32*(5), 577–585. 10.1002/pds.5591 36585827 10.1002/pds.5591 PMC10073250

[CR35] Roland, C. L., Lake, J., & Oderda, G. M. (2016). Prevalence of prescription opioid misuse/abuse as determined by International Classification of Diseases codes: A systematic review. *Journal of Pain & Palliative Care Pharmacotherapy,**30*(4), 258–268. 10.1080/15360288.2016.1231739 27802072 10.1080/15360288.2016.1231739

[CR36] Rowe, C., Vittinghoff, E., Santos, G. M., Behar, E., Turner, C., & Coffin, P. O. (2017). Performance measures of diagnostic codes for detecting opioid overdose in the emergency department. *Academic Emergency Medicine,**24*(4), 475–483. 10.1111/acem.13121 27763703 10.1111/acem.13121

[CR37] Sarrazin, M. S., & Rosenthal, G. E. (2012). Finding pure and simple truths with administrative data. *JAMA,**307*(13), 1433–1435. 10.1001/jama.2012.404 10.1001/jama.2012.404 22474208

[CR38] Slavova, S., Bunn, T. L., & Talbert, J. (2014). Drug overdose surveillance using hospital discharge data. *Public Health Reports,**129*(5), 437–445. 10.1177/003335491412900507 25177055 10.1177/003335491412900507 PMC4116371

[CR39] UNODC. (2022). *World Drug Report 2022 [Internet]*. United Nations Office on Drugs and Crime. Retrieved 2024 Jan 8 from https://www.unodc.org/res/wdr2022/MS/WDR22_Booklet_1.pdf

[CR40] Velagapudi, V., & Sethi, R. (2023). Illicit non-pharmaceutical fentanyl and its analogs: A short review of literature. *Kansas Journal of Medicine, 16*, 25–27. 10.17161/kjm.vol16.18555 10.17161/kjm.vol16.18555 PMC9872502 36703950

[CR41] Vivolo-Kantor, A., Pasalic, E., Liu, S., Martinez, P. D., & Gladden, R. M. (2021). Defining indicators for drug overdose emergency department visits and hospitalisations in ICD-10-CM coded discharge data. *Injury Prevention,**27*(S1), i56–i61. 10.1136/injuryprev-2019-043521 33674334 10.1136/injuryprev-2019-043521 PMC7948191

[CR42] Walker, R. L., Hennessy, D. A., Johansen, H., Sambell, C., Lix, L., & Quan, H. (2012). Implementation of ICD-10 in Canada: How has it impacted coded hospital discharge data? *BMC Health Services Research,**12*, 149. 10.1186/1472-6963-12-149 22682405 10.1186/1472-6963-12-149 PMC3411494

[CR43] Ward, R., Obeid, J. S., Jennings, L., Szwast, E., Hayes, W. G., Pipaliya, R., Bailey, C., Faul, S., Polyak, B., Baker, G. H., McCauley, J. L., & Lenert, L. A. (2023). Enhanced phenotypes for identifying opioid overdose in emergency department visit electronic health record data. *Jamia Open, 6*(3), ooad081. 10.1093/jamiaopen/ooad081 10.1093/jamiaopen/ooad081 PMC10938047 38486917

[CR44] Whiting, P. F., Rutjes, A. W., Westwood, M. E., Mallett, S., Deeks, J. J., Reitsma, J. B., Leeflang, M. M., Sterne, J. A., & Bossuyt, P. M. (2011). QUADAS-2: A revised tool for the quality assessment of diagnostic accuracy studies. *Annals of Internal Medicine,**155*(8), 529–536. 10.7326/0003-4819-155-8-201110180-00009 22007046 10.7326/0003-4819-155-8-201110180-00009

[CR45] Xiang, Y., Zhao, W., Xiang, H., & Smith, G. A. (2012). ED visits for drug-related poisoning in the United States, 2007. *American Journal of Emergency Medicine,**30*(2), 293–301. 10.1016/j.ajem.2010.11.031 21367556 10.1016/j.ajem.2010.11.031

[CR46] Zhu, V. J., Lenert, L. A., Barth, K. S., Simpson, K. N., Li, H., Kopscik, M., & Brady, K. T. (2022). Automatically identifying opioid use disorder in non-cancer patients on chronic opioid therapy. *Health Informatics Journal,**28*(2), 14604582221107808. 10.1177/14604582221107808 35726687 10.1177/14604582221107808 PMC10826411

