# Supplementary material for: Sensitivity and specificity of International Classification of Diseases algorithms (ICD-9 and ICD-10) used to identify opioid-related overdose cases: A systematic review and an example of estimation using Bayesian latent class models in the absence of gold standards
Source: Can J Public Health. 2024 Jul 31;115(5):770–83. doi: 10.17269/s41997-024-00915-4 (PMC11535208; doi:10.17269/s41997-024-00915-4)
Supplement: Supplementary file 2 — Supplementary file2 (DOCX 30 KB) [file 41997_2024_915_MOESM2_ESM.docx]

**Title:** Sensitivity and specificity of International Classification of Diseases algorithms (ICD-9 and ICD-10) Used to Identify Opioid-Related Overdose Cases: a systematic review and an example of estimation using Bayesian Latent Class Models in the absence of gold standards

**Journal Name:** Canadian Journal of Public Health

**Online Resource 2: Literature Search Strategy**

1. ***Initial search***

The initial search was executed by SET on May 6, 2023.

Database(s): **Ovid MEDLINE(R)**and **Embase.**

Search Strategy:

| **#** | **Searches** |
| --- | --- |
| 1 | "Sensitivity and Specificity"/ or Predictive Value of Tests/ or validation studies as topic/ |
| 2 | (Sensitivit* or specificit* or reliabilit* or validit* or accura*or precision* or predictive value* or negative predictive* or positive predictive* or validation stud* or performance* or consistence* or particularit*).ab,kf,kw,ti. |
| 3 | 1 or 2 |
| 4 | Opiate Overdose/ or Drug Overdose/ or mortality/ or "cause of death"/ or "Drug-Related Side Effects and Adverse Reactions"/ or poisoning/ |
| 5 | (Overdos* or toxic or toxicity or death* or mortality or mortalities or poisoning).ab,kf,kw,ti. |
| 6 | 4 or 5 |
| 7 | exp analgesics, opioid/ |
| 8 | (opioid* or opiac* or opiate*or Heroin*or cocain* or methadone* or methadose* or methex* or fentanyl* or analgesic* Tramadol* or Remifentanil or Sufentanil or Oxycodone* or Pentazocine or Opium* or Morphine* or Meperidine or Codein* or Buprenorphine or methamphetamin*).ab,kf,kw,ti. |
| 9 | 7 or 8 |
| 10 | substance-related disorders/ or amphetamine-related disorders/ or cocaine-related disorders/ or narcotic-related disorders/ or exp opioid-related disorders/ or substance abuse, intravenous/ or substance abuse, oral/ |
| 11 | (" substance use" or ((substance* or amphetamine or cocaine or narcotic or opioid*) adj2 (abuse or misuse or overuse or disorder* or "over utilisation" or "over utilization"))).ab,kf,kw,ti. |
| 12 | 10 or 11 |
| 13 | 9 or 12 |
| 14 | "International Classification of Diseases"/ |
| 15 | ("ICD-9*" or "ICD-10*" or "International Classification of Disease*" or icd9* or icd10* or "ICD*").ab,kf,kw,ti. |
| 16 | 14 or 15 |
| 17 | mathematical concepts/ or exp algorithms/ |
| 18 | ("Algorithm*" or arithmetic or calculation or mathematical or machine learning).ab,kf,kw,ti. |
| 19 | 17 or 18 |
| 20 | Classification/ |
| 21 | ("classifi*" or indexing or indexation or archiv* or categorization* or categorisation* or typolog* or nomenclature*).ab,kf,kw,ti. |
| 22 | 20 or 21 |
| 23 | 16 or 19 or 22 |
| 24 | 3 and 6 and 13 and 23 |

*Results:*

A total of 753 studies (MEDLINE: 227, Embase: 526) were imported for screening.

1. ***Second search***

The second search was executed by FINM on December 13, 2023 and reconsidered the algorithms of the initial search, adding a few keywords indicated in bold (see lines 4, 5 and 8).

Database(s): **Ovid MEDLINE(R) ALL**1946 to December 07, 2023

Search Strategy:

| **#** | **Searches** | **Results** |
| --- | --- | --- |
| 1 | "Sensitivity and Specificity"/ or Predictive Value of Tests/ or validation studies as topic/ | 560372 |
| 2 | (Sensitivit* or specificit* or reliabilit* or validit* or accura*or precision* or predictive value* or negative predictive* or positive predictive* or validation stud* or performance* or consistence* or particularit*).ab,kf,kw,ti. | 2945140 |
| 3 | 1 or 2 | 3247641 |
| 4 | **Opioid Overdose/ or** Opiate Overdose/ or Drug Overdose/ or mortality/ or "cause of death"/ or "Drug-Related Side Effects and Adverse Reactions"/ or poisoning/ **or "Opioid Use Disorder"/** | 191606 |
| 5 | (Overdos* or toxic or toxicity or death* or mortality or mortalities or poisoning **or** **disorder***).ab,kf,kw,ti. | 3907047 |
| 6 | 4 or 5 | 3974369 |
| 7 | exp analgesics, opioid/ | 138004 |
| 8 | (opioid* or opiac* or opiate*or Heroin*or cocain* or methadone* or methadose* or methex* or fentanyl* or analgesic* Tramadol* or Remifentanil or Sufentanil or Oxycodone* or Pentazocine or Opium* or Morphine* or Meperidine or Codein* or Buprenorphine or methamphetamin* **or** **Naloxone**).ab,kf,kw,ti. | 212069 |
| 9 | 7 or 8 | 243469 |
| 10 | substance-related disorders/ or amphetamine-related disorders/ or cocaine-related disorders/ or narcotic-related disorders/ or exp opioid-related disorders/ or substance abuse, intravenous/ or substance abuse, oral/ | 162011 |
| 11 | (" substance use" or ((substance* or amphetamine or cocaine or narcotic or opioid*) adj2 (abuse or misuse or overuse or disorder* or "over utilisation" or "over utilization"))).ab,kf,kw,ti. | 93701 |
| 12 | 10 or 11 | 206053 |
| 13 | 9 or 12 | 398063 |
| 14 | "International Classification of Diseases"/ | 9524 |
| 15 | ("ICD-9*" or "ICD-10*" or "International Classification of Disease*" or icd9* or icd10* or "ICD*").ab,kf,kw,ti. | 63520 |
| 16 | 14 or 15 | 67154 |
| 17 | mathematical concepts/ or exp algorithms/ | 450377 |
| 18 | ("Algorithm*" or arithmetic or calculation or mathematical or machine learning).ab,kf,kw,ti. | 666544 |
| 19 | 17 or 18 | 917012 |
| 20 | Classification/ | 10609 |
| 21 | ("classifi*" or indexing or indexation or archiv* or categorization* or categorisation* or typolog* or nomenclature*).ab,kf,kw,ti. | 881381 |
| 22 | 20 or 21 | 886574 |
| 23 | 16 or 19 or 22 | 1735832 |
| 24 | 3 and 6 and 13 and 23 | 916 |

Database(s): **Embase** 1974 to 2023 December 08

Search Strategy:

| **#** | **Searches** | **Results** |
| --- | --- | --- |
| 1 | "Sensitivity and Specificity"/ or Predictive Value of Tests/ or validation studies as topic/ | 693153 |
| 2 | (Sensitivit* or specificit* or reliabilit* or validit* or accura*or precision* or predictive value* or negative predictive* or positive predictive* or validation stud* or performance* or consistence* or particularit*).ab,kf,kw,ti. | 3623335 |
| 3 | 1 or 2 | 3862584 |
| 4 | **Opioid Overdose/ or** Opiate Overdose/ or Drug Overdose/ or mortality/ or "cause of death"/ or "Drug-Related Side Effects and Adverse Reactions"/ or poisoning/ **or "Opioid Use Disorder"/** | 1389495 |
| 5 | (Overdos* or toxic or toxicity or death* or mortality or mortalities or poisoning **or** **disorder***).ab,kf,kw,ti. | 5361597 |
| 6 | 4 or 5 | 5845268 |
| 7 | exp analgesics, opioid/ | 408506 |
| 8 | (opioid* or opiac* or opiate*or Heroin*or cocain* or methadone* or methadose* or methex* or fentanyl* or analgesic* Tramadol* or Remifentanil or Sufentanil or Oxycodone* or Pentazocine or Opium* or Morphine* or Meperidine or Codein* or Buprenorphine or methamphetamin* **or Naloxone**).ab,kf,kw,ti. | 284709 |
| 9 | 7 or 8 | 487123 |
| 10 | substance-related disorders/ or amphetamine-related disorders/ or cocaine-related disorders/ or narcotic-related disorders/ or exp opioid-related disorders/ or substance abuse, intravenous/ or substance abuse, oral/ | 173198 |
| 11 | (" substance use" or ((substance* or amphetamine or cocaine or narcotic or opioid*) adj2 (abuse or misuse or overuse or disorder* or "over utilisation" or "over utilization"))).ab,kf,kw,ti. | 124898 |
| 12 | 10 or 11 | 236905 |
| 13 | 9 or 12 | 660193 |
| 14 | "International Classification of Diseases"/ | 18221 |
| 15 | ("ICD-9*" or "ICD-10*" or "International Classification of Disease*" or icd9* or icd10* or "ICD*").ab,kf,kw,ti. | 134248 |
| 16 | 14 or 15 | 139847 |
| 17 | mathematical concepts/ or exp algorithms/ | 608407 |
| 18 | ("Algorithm*" or arithmetic or calculation or mathematical or machine learning).ab,kf,kw,ti. | 821285 |
| 19 | 17 or 18 | 1085347 |
| 20 | Classification/ | 342130 |
| 21 | ("classifi*" or indexing or indexation or archiv* or categorization* or categorisation* or typolog* or nomenclature*).ab,kf,kw,ti. | 1228905 |
| 22 | 20 or 21 | 1439976 |
| 23 | 16 or 19 or 22 | 2472475 |
| 24 | 3 and 6 and 13 and 23 | 1582 |
