# Supplementary material for: Sensitivity and specificity of International Classification of Diseases algorithms (ICD-9 and ICD-10) used to identify opioid-related overdose cases: A systematic review and an example of estimation using Bayesian latent class models in the absence of gold standards
Source: Can J Public Health. 2024 Jul 31;115(5):770–83. doi: 10.17269/s41997-024-00915-4 (PMC11535208; doi:10.17269/s41997-024-00915-4)
Supplement: Supplementary file 3 — Supplementary file3 (DOCX 31 KB) [file 41997_2024_915_MOESM3_ESM.docx]

**Title:** Sensitivity and specificity of International Classification of Diseases algorithms (ICD-9 and ICD-10) Used to Identify Opioid-Related Overdose Cases: a systematic review and an example of estimation using Bayesian Latent Class Models in the absence of gold standards

**Journal Name:** Canadian Journal of Public Health

**Online Resource 3: QUADAS-2 instructions used for assessing bias and applicability.**

The instructions for the assessment of study bias and applicability reported below were adapted from the work by McGrew et al. (1) which examined the validity of International Classification of Diseases Ninth or Tenth Revision (ICD-9 or ICD-10) codes in identifying illicit drug use target conditions using medical record data as a reference standard.

Bias Assessment with QUADAS-2

QUADAS-2 includes the following four domains: (1) patient selection, (2) index test, (3) the reference standard, and (4) flow of patients and timing of the index test and reference standard. For each domain, the risk of bias is scored as “low”, “high”, or “unclear”. For the first three domains, applicability is evaluated and scored as “low”, “high”, or “unclear”.

Risk of Bias Judgements

If all signaling questions for a domain are answered “yes” then risk of bias can be considered “low”.

If any signaling question is answered “no” then the risk of bias should be considered “high”.

The “unclear” category should be used only when insufficient data are reported to permit a judgment.

Applicability Judgements

Applicability judgements should be made considering the systematic review research questions:

This systematic review aims to answer the following questions based on studies using medical record abstraction as a reference standard:

1. What are the sensitivity and specificity of ICD-9 or ICD-10 algorithms to identify OOD-related events?

2. Which algorithm (ICD-9 or ICD-10) performs better in terms of sensitivity and specificity to detect opioid-related poisonings?

***DOMAIN 1: PATIENT SELECTION***

**Free-text question: Describe methods of patient selection.**

- Describe how patients were selected (e.g., consecutive, random sample) and the criteria for selection. Where were patients selected from (e.g., hospitals in a geographic region)?

**Signaling question 1: Was a consecutive or random sample of patients enrolled?**

- Select ‘Yes’ if consecutive or random sampling was used. Also select ‘yes’ if ‘consecutive sampling’ was not explicitly stated but was described (i.e. ‘all patients hospitalized between [date] and [date] were included’).
- Select ‘No’ if non-consecutive or convenience sampling was used.
- Select ‘Unclear’ if insufficient information is reported.

**Signaling question 2: Was a case-control design avoided?**

**Signaling question 3: Did the study avoid inappropriate exclusions?**

- Select ‘Yes’ if all eligible patients were included or if exclusions are reasonable (describe).
- Select ‘No’ if inappropriate exclusions are identified such as patients who are “difficult to diagnose” or patients with “red flags” for the target condition (i.e., those easier to diagnose)
- Select ‘Unclear’ if insufficient information is reported.

**Risk of bias: Could the selection of patients have introduced bias?**

- If all signaling questions are answered “yes” then risk of bias can be considered “low”.
- If any signaling question is answered “no” then the risk of bias should be considered “high”.
- The “unclear” category should be used only when insufficient data are reported to permit a judgement.

**Applicability: Are there concerns that the included patients and setting do not match the review question?**

Review the systematic review research questions on page 1.

***DOMAIN 2: INDEX TEST (ICD-9 or ICD-10 CODES/ALGORITHMS)***

**Free-text question: Describe the index test and how it was conducted and interpreted.**

- What was the source of the ICD codes (e.g., hospital discharge data, outpatient data)?
- What ICD code position was considered (i.e., primary only or primary and secondary diagnoses)?

**Signaling question 1: Were the index test results interpreted without knowledge of the results of the reference standard?**

- Select ‘Yes’ if the ICD codes were interpreted without knowledge of the results of the reference standard diagnosis.
- Select ‘No’ if it was reported that ICD codes were interpreted with knowledge of the results of the reference standard diagnosis.
- Select ‘Unclear’ if insufficient information is reported.

**Signaling question 2: If a threshold was used, was it pre-specified?**

This signaling question was not considered, as the outcome of the review was binary (presence/absence of opioid overdose event).

**Risk of Bias: Could the conduct or interpretation of the index test have introduced bias?**

- If all signaling questions are answered “yes” then risk of bias can be considered “low”.
- If any signaling question is answered “no” then the risk of bias should be considered “high”.
- The “unclear” category should be used only when insufficient data are reported to permit a judgement.

**Applicability: Are there concerns that the index test, its conduct, or interpretation differ from the review question?**

If index test methods vary from those specified in the review question, there may be concerns regarding applicability.

***DOMAIN 3: REFERENCE STANDARD***

**Free-text question: Describe the reference standard and how it was conducted and interpreted.**

- What was the reference standard used?
- Who read the reference standard (e.g., nurse, physician, or research assistant)? What was their training/experience?
- If more than one person has read the reference standard, what was the measure of interrater agreement (e.g. kappa)?
- Was the reference standard applied according to pre-established standardized procedures (e.g., the use of a standardized medical record abstraction form in case of chart review)?
- How were indeterminate results or discrepancies treated?

**Signaling question 1: Is the reference standard likely to correctly classify the target condition?**

- Select ‘Yes’ if
  - The reference standard was applied according to pre-established standardized procedures (e.g., the use of a standardized medical record abstraction form), or the criteria defining a case were clearly indicated, AND
  - Measures of agreement were calculated if more than one person read the reference standard, AND
  - Reader(s) received some sort of training or possessed the required credentials (e.g., nurse, physician)
- Select ‘No’ if
  - the authors reported reference standard was not applied according to pre-established standardized procedures (e.g., lack of use of a standardized medical record abstraction form in case of chart review), or the criteria defining a case were not clearly indicated, OR
  - more than one person read the reference standard but agreement between readers was not assessed, OR
  - readers had no training/clinical expertise.
- Select ‘Unclear’ if insufficient information is reported (e.g., no information was reported on interrater agreement between readers).

**Signaling question 2: Were the reference standard results interpreted without knowledge of the results of the index test?**

- Select ‘Yes’ if it was reported that the readers of the reference standard were blinded to the ICD codes.
- Select ‘No’ if it was reported that the readers of the reference standard were not blinded to the ICD codes.
- Select ‘Unclear’ if insufficient information is reported.

**Risk of Bias: Could the reference standard, its conduct, or its interpretation have introduced bias?**

- If all signaling questions are answered “yes” then risk of bias can be considered “low”.
- If any signaling question is answered “no” then the risk of bias should be considered “high”.
- The “unclear” category should be used only when insufficient data are reported to permit a judgement.

**Applicability: Are there concerns that the target condition as defined by the reference standard does not match the question?**

Review the systematic review research questions on page 1. The target condition defined by the reference standard may differ from the target condition specified in the review question.

***DOMAIN 4: FLOW AND TIMING***

**Free-text question: Describe any patients who did not receive the index test(s) and/or reference standard or who were excluded from the 2x2 table (refer to the flow diagram):**

- Was a flow diagram included?
- If participants were excluded from the final 2x2 table, were the reasons for this exclusion explained? Were any characteristics of excluded participants described?

**Signaling question 1: Was there an appropriate interval between index test and reference standard? (**i.e., was the time period between the medical record documentation and the selection of the ICD codes short enough to be reasonably sure that the target condition status did not change?)

- Select ‘Yes’ if the medical record information used to make the reference standard diagnosis was the same as the medical record information used at the time of coding (i.e., the information should be from the same healthcare encounter).
- Select ‘No’ if the medical record information used to make the reference standard diagnosis was NOT the same as the medical record information used at the time of coding (e.g., medical record information from past or future hospitalizations was considered part of the reference standard).
- Select ‘Unclear’ if insufficient information is reported.

**Signaling question 2: Did all patients receive a reference standard?**

**Signaling question 3: Did patients receive the same reference standard?**

- **S**elect ‘Yes’ if
  - The reference standard was applied by one person, OR
  - The reference standard was applied by more than one person and agreement between readers was quantified (e.g., percent agreement, the kappa statistic) and reasonably high (e.g., kappa statistic of substantial agreement – almost perfect agreement)
- Select ‘No’ if the reference standard was applied by more than one person and
  - the investigators did not assess agreement between readers, OR
  - the measured interrater agreement was inadequate (e.g., kappa statistic of moderate agreement or lower)
- Select ‘Unclear’ if insufficient information is reported (e.g., no information on interrater agreement was reported).

**Signaling question 4: Were all patients included in the analysis?**

- **S**elect ‘Yes’ if the number of patients enrolled is the same as the number of patients included in the 2x2 table of results.
- Select ‘No’ if the number of patients enrolled differs from the number of patients included in the 2x2 table of results.
- Select ‘Unclear’ if insufficient information is reported (e.g., no information on how the final validation study population was achieved).

**Risk of Bias: Could the patient flow have introduced bias?**

- If all signaling questions are answered “yes” then risk of bias can be considered “low”.
- If any signaling question is answered “no” then the risk of bias should be considered “high”.
- The “unclear” category should be used only when insufficient data are reported to permit a judgement.

**Reference**

1. McGrew KM, Homco JB, Garwe T, et al. Validity of International Classification of Diseases codes in identifying illicit drug use target conditions using medical record data as a reference standard: A systematic review. Drug Alcohol Depend. 2020;208:107825.
