# Supplementary material for: Sensitivity and specificity of International Classification of Diseases algorithms (ICD-9 and ICD-10) used to identify opioid-related overdose cases: A systematic review and an example of estimation using Bayesian latent class models in the absence of gold standards
Source: Can J Public Health. 2024 Jul 31;115(5):770–83. doi: 10.17269/s41997-024-00915-4 (PMC11535208; doi:10.17269/s41997-024-00915-4)
Supplement: Supplementary file 4 — Supplementary file4 (DOCX 22 KB) [file 41997_2024_915_MOESM4_ESM.docx]

**Title:** Sensitivity and specificity of International Classification of Diseases algorithms (ICD-9 and ICD-10) Used to Identify Opioid-Related Overdose Cases: a systematic review and an example of estimation using Bayesian Latent Class Models in the absence of gold standards

**Journal Name:** Canadian Journal of Public Health

**Online Resource 4:** **R codes run in JAGS to estimate sensitivity and specificity of ICD-10 algorithms and coroner's report review (CRR) in diagnosing prescription opioid-related deaths among all drug- or alcohol-related deaths in Ontario using Bayesian latent class models.**

**LATENT CLASS MODEL ASSUMING CONDITIONAL INDEPENDENCE BETWEEN ICD AND CRR**

1. **Model using scenario 1 of priors**

model{

#=== LIKELIHOOD ===#

n[1:4] ~ dmulti(p[1:4], n)

p[1] <- Prev*Se_CRR*Se_ICD + (1-Prev)*(1-Sp_CRR)*(1-Sp_ICD)

p[2] <- Prev*Se_CRR*(1-Se_ICD) + (1-Prev)*(1-Sp_CRR)*Sp_ICD

p[3] <- Prev*(1-Se_CRR)*Se_ICD + (1-Prev)*Sp_CRR*(1-Sp_ICD)

p[4] <- Prev*(1-Se_CRR)*(1-Se_ICD) + (1-Prev)*Sp_CRR*Sp_ICD

#=== PRIOR ===#

Prev ~ dunif(0.00, 0.102) ## Uniform prior for prevalence

Se_CRR ~ dunif(0.75, 1.00) ## Uniform prior for CRR Se

Sp_CRR ~ dunif(0.90, 1.00) ## Uniform prior for CRR Sp

Se_ICD ~ dunif(0.75, 1.00) ## Uniform prior for ICD Se

Sp_ICD ~ dunif(0.90, 1.00) ## Uniform prior for ICD Sp

}

1. **Model using scenario 2 of priors**

model{

#=== LIKELIHOOD ===#

n[1:4] ~ dmulti(p[1:4], n)

p[1] <- Prev*Se_CRR*Se_ICD + (1-Prev)*(1-Sp_CRR)*(1-Sp_ICD)

p[2] <- Prev*Se_CRR*(1-Se_ICD) + (1-Prev)*(1-Sp_CRR)*Sp_ICD

p[3] <- Prev*(1-Se_CRR)*Se_ICD + (1-Prev)*Sp_CRR*(1-Sp_ICD)

p[4] <- Prev*(1-Se_CRR)*(1-Se_ICD) + (1-Prev)*Sp_CRR*Sp_ICD

#=== PRIOR ===#

Prev ~ dunif(0.00, 0.102) ## Uniform prior for prevalence

Se_CRR ~ dunif(0.00, 1.00) ## Uniform prior for CRR Se

Sp_CRR ~ dunif(0.00, 1.00) ## Uniform prior for CRR Sp

Se_ICD ~ dunif(0.00, 1.00) ## Uniform prior for ICD Se

Sp_ICD ~ dunif(0.00, 1.00) ## Uniform prior for ICD Sp

}
